# Supplementary material for: Meta-Analysis and Gene Set Enrichment Relative to ER Status Reveal Elevated Activity of MYC and E2F in the “Basal” Breast Cancer Subgroup
Source: PLoS One. 2009 Mar 9;4(3):e4710. doi: 10.1371/journal.pone.0004710 (PMC2650420; doi:10.1371/journal.pone.0004710)
Supplement: Dataset S1 — GSEA results from first screen. The file contains a zipped archive of the HTML results of the second GSEA screen. Reports for enriched or depleted datasets may be accessed from the “index.html” link within the my_analysis.GseaPreranked.1219975950625.rpt.zip ZIP archive. Enrichment plots are available for each of the 50 most enriched and depleted gene sets. (13.32 MB ZIP) [file pone.0004710.s001.zip › my_analysis.GseaPreranked.1219975950625.rpt.zip!//CHR17Q23.html]

Details for gene set CHR17Q23[GSEA]

|  || Dataset | zSco.ERneg.to.ERpos\_collapsed |
| Phenotype | NoPhenotypeAvailable |
| Upregulated in class | na\_neg |
| GeneSet | CHR17Q23 |
| Enrichment Score (ES) | -0.387491 |
| Normalized Enrichment Score (NES) | -1.5935626 |
| Nominal p-value | 0.0028985508 |
| FDR q-value | 0.13087752 |
| FWER p-Value | 0.999 |
Table: GSEA Results Summary

  

Fig 1: Enrichment plot: CHR17Q23      
 Profile of the Running ES Score & Positions of GeneSet Members on the Rank Ordered List

  

| PROBE | GENE SYMBOL | GENE\_TITLE | RANK IN GENE LIST | RANK METRIC SCORE | RUNNING ES | CORE ENRICHMENT || 1 | KCNJ2 |  |  | 296 | 4.948 | 0.0360 | No |
| 2 | TMEM100 |  |  | 1400 | 3.039 | -0.0113 | No |
| 3 | LIMD2 |  |  | 2333 | 2.305 | -0.0544 | No |
| 4 | LPO |  |  | 2374 | 2.271 | -0.0307 | No |
| 5 | ICAM2 |  |  | 2624 | 2.132 | -0.0243 | No |
| 6 | TK1 |  |  | 2730 | 2.076 | -0.0078 | No |
| 7 | SOX9 |  |  | 2768 | 2.058 | 0.0136 | No |
| 8 | CD79B |  |  | 3075 | 1.896 | 0.0129 | No |
| 9 | RNF126P1 |  |  | 3290 | 1.797 | 0.0180 | No |
| 10 | ERN1 |  |  | 3513 | 1.701 | 0.0213 | No |
| 11 | KCNH6 |  |  | 3875 | 1.563 | 0.0125 | No |
| 12 | TRIM25 |  |  | 4161 | 1.448 | 0.0080 | No |
| 13 | WDR68 |  |  | 4390 | 1.360 | 0.0069 | No |
| 14 | PRR11 |  |  | 5353 | 1.025 | -0.0535 | No |
| 15 | EPX |  |  | 6278 | 0.722 | -0.1147 | No |
| 16 | NACAL |  |  | 7333 | 0.371 | -0.1897 | No |
| 17 | C17ORF60 |  |  | 7559 | 0.286 | -0.2033 | No |
| 18 | TBX2 |  |  | 7703 | 0.228 | -0.2114 | No |
| 19 | CA4 |  |  | 7906 | 0.144 | -0.2249 | No |
| 20 | KCNJ16 |  |  | 7928 | 0.136 | -0.2249 | No |
| 21 | SCN4A |  |  | 8152 | 0.055 | -0.2411 | No |
| 22 | FLJ11710 |  |  | 8218 | 0.030 | -0.2456 | No |
| 23 | MRC2 |  |  | 8705 | -0.172 | -0.2802 | No |
| 24 | PTRH2 |  |  | 8919 | -0.270 | -0.2931 | No |
| 25 | ACE |  |  | 8977 | -0.304 | -0.2938 | No |
| 26 | PPM1D |  |  | 9078 | -0.350 | -0.2972 | No |
| 27 | TUBD1 |  |  | 9220 | -0.414 | -0.3030 | No |
| 28 | USP32 |  |  | 9362 | -0.490 | -0.3078 | No |
| 29 | GALK1 |  |  | 9378 | -0.495 | -0.3031 | No |
| 30 | PECAM1 |  |  | 10138 | -0.925 | -0.3494 | No |
| 31 | RPS6KB1 |  |  | 10180 | -0.944 | -0.3414 | No |
| 32 | MRPS7 |  |  | 10604 | -1.248 | -0.3586 | No |
| 33 | PRKAR1A |  |  | 10989 | -1.556 | -0.3692 | Yes |
| 34 | TLK2 |  |  | 11040 | -1.608 | -0.3540 | Yes |
| 35 | CYB561 |  |  | 11450 | -1.990 | -0.3614 | Yes |
| 36 | C17ORF71 |  |  | 11468 | -2.004 | -0.3390 | Yes |
| 37 | SMARCD2 |  |  | 11483 | -2.016 | -0.3164 | Yes |
| 38 | ABC1 |  |  | 11545 | -2.084 | -0.2964 | Yes |
| 39 | CCDC47 |  |  | 11631 | -2.150 | -0.2775 | Yes |
| 40 | VEZF1 |  |  | 11771 | -2.295 | -0.2609 | Yes |
| 41 | LOC51136 |  |  | 11893 | -2.433 | -0.2414 | Yes |
| 42 | CCDC44 |  |  | 11939 | -2.484 | -0.2155 | Yes |
| 43 | FTSJ3 |  |  | 12050 | -2.637 | -0.1927 | Yes |
| 44 | CUEDC1 |  |  | 12171 | -2.804 | -0.1688 | Yes |
| 45 | LYK5 |  |  | 12222 | -2.875 | -0.1387 | Yes |
| 46 | DDX42 |  |  | 12247 | -2.907 | -0.1062 | Yes |
| 47 | TANC2 |  |  | 12374 | -3.057 | -0.0797 | Yes |
| 48 | BCAS3 |  |  | 12404 | -3.106 | -0.0453 | Yes |
| 49 | RNF43 |  |  | 12408 | -3.113 | -0.0089 | Yes |
| 50 | TEX2 |  |  | 12507 | -3.273 | 0.0223 | Yes |
| 51 | PSMC5 |  |  | 12531 | -3.303 | 0.0595 | Yes |
Table: GSEA details [plain text format]

  

Fig 2: CHR17Q23: Random ES distribution      
 Gene set null distribution of ES for **CHR17Q23**
